# Explainable Machine Learning Techniques to Predict Muscle Injuries in Professional Soccer Players through Biomechanical Analysis

**DOI:** 10.3390/s24010119

**Published:** 2023-12-25

**Authors:** Mailyn Calderón-Díaz, Rony Silvestre Aguirre, Juan P. Vásconez, Roberto Yáñez, Matías Roby, Marvin Querales, Rodrigo Salas

**Affiliations:** 1 Faculty of Engineering, Universidad Andres Bello, Santiago 7550196, Chile; juan.vasconez@unab.cl; 2 Ph.D. Program in Health Sciences and Engineering, Universidad de Valparaiso, Valparaiso 2362735, Chile; 3 Millennium Institute for Intelligent Healthcare Engineering (iHealth), Valparaiso 2362735, Chile; 4 Laboratorio de Biomecánica, Centro de Innovación Clínica MEDS, Santiago 7691236, Chile; rony.silvestre@meds.cl (R.S.A.); roberto.yanez@meds.cl (R.Y.); matias.roby@meds.cl (M.R.); 5 School of Medical Technology, Universidad de Valparaiso, Valparaiso 2362735, Chile; marvin.querales@uv.cl; 6 School of Biomedical Engineering, Universidad de Valparaiso, Valparaiso 2362735, Chile

**Keywords:** machine learning explainability, sport medicine, hamstring injuries, soccer player, XGBoost

## Abstract

There is a significant risk of injury in sports and intense competition due to the demanding physical and psychological requirements. Hamstring strain injuries (HSIs) are the most prevalent type of injury among professional soccer players and are the leading cause of missed days in the sport. These injuries stem from a combination of factors, making it challenging to pinpoint the most crucial risk factors and their interactions, let alone find effective prevention strategies. Recently, there has been growing recognition of the potential of tools provided by artificial intelligence (AI). However, current studies primarily concentrate on enhancing the performance of complex machine learning models, often overlooking their explanatory capabilities. Consequently, medical teams have difficulty interpreting these models and are hesitant to trust them fully. In light of this, there is an increasing need for advanced injury detection and prediction models that can aid doctors in diagnosing or detecting injuries earlier and with greater accuracy. Accordingly, this study aims to identify the biomarkers of muscle injuries in professional soccer players through biomechanical analysis, employing several ML algorithms such as decision tree (DT) methods, discriminant methods, logistic regression, naive Bayes, support vector machine (SVM), K-nearest neighbor (KNN), ensemble methods, boosted and bagged trees, artificial neural networks (ANNs), and XGBoost. In particular, XGBoost is also used to obtain the most important features. The findings highlight that the variables that most effectively differentiate the groups and could serve as reliable predictors for injury prevention are the maximum muscle strength of the hamstrings and the stiffness of the same muscle. With regard to the 35 techniques employed, a precision of up to 78% was achieved with XGBoost, indicating that by considering scientific evidence, suggestions based on various data sources, and expert opinions, it is possible to attain good precision, thus enhancing the reliability of the results for doctors and trainers. Furthermore, the obtained results strongly align with the existing literature, although further specific studies about this sport are necessary to draw a definitive conclusion.

## 1. Introduction

In sports and intense competition, there is a substantial risk of sustaining injuries arising from the demanding physical and psychological pressures. These injuries impact the athletes and have ripple effects on coaches, sponsors, teams, and clubs, compounded by the substantial medical expenses involved [1,2,3,4]. In sports like soccer, lower extremity injuries claim a significant share, amounting to 92% of the total injury count [5,6,7,8]. The sport’s physical demands and specific characteristics contribute significantly to this heightened injury incidence [5,9]. Notably, hamstring strain injuries (HSIs) represent the most prevalent injury in football, making up 12% of all reported injuries. Alarmingly, the recurrence rate for this injury ranges between 12% and 41% within the first year of returning to the sport [1,2]. Despite this, a recent meta-analysis revealed that seemingly, high-level soccer teams do not implement any injury prevention protocols [10].

Considering the multi-factorial nature of injuries, identifying the most crucial risk factors and their interplay, and devising effective prevention strategies present considerable challenges [11,12]. In response, attention has turned toward the potential of artificial intelligence tools [13]. By leveraging expansive datasets and predictive models, healthcare professionals can diagnose, predict, and treat their patients with heightened confidence [14]. Examples include forecasting post-cardiac surgery complications [15], predicting ICU mortality due to COVID-19 [16], anticipating outcomes following knee surgery [17], diagnosing pathologies in lumbar spine MRIs [18], and foreseeing surgical risks [19], among others. However, many machine learning (ML) models often lack user-friendliness for individuals interacting with them. Achieving an understanding, termed explainability or interpretability, is critical for human users to comprehend and trust the machine’s decision-making process [20]. While the development of explainable models is relatively recent, their value in interpreting ML models has been acknowledged by various experts [21,22]. Notably, decision trees have emerged as prominent tools in healthcare, as many health professionals are already familiar with them in their practice, considering that clinical, serological, or radiological data often underpin similar medical decisions [14]. With this in mind, this study aims to determine the biomarkers of hamstring injuries in professional soccer players through biomechanical analysis using machine learning techniques, emphasizing a certain level of explainability. To achieve this, this study will implement 35 machine learning classification algorithms, explicitly focusing on applying the XGboost (extra gradient boosting) technique. XGboost represents an implementation of decision trees with gradient boosting, offering the added advantage of automatically providing estimates of the feature importance within a trained predictive model [23]. By implementing machine learning algorithms, we aim to not only improve accuracy in predicting results and player performance but also provide clear and understandable insights for coaches, players, and fans. This novel approach focuses not only on optimizing results but also on the transparency and understanding of the decisions made by the model, thus opening up new possibilities for the effective application of machine learning in the field of soccer.

## 2. State of the Art

Thigh muscles are commonly injured in soccer players due to the movement pattern in performing rapid accelerations and decelerations, causing the muscles to overstretch [11]. Risk factors for hamstring injuries are a matter of debate, and many studies have been conducted to investigate possible predictors. A systematic review evaluating high-quality prospective studies in soccer players recognized previous injury as the only significant risk factor [11,24]. Furthermore, the authors concluded that, among other factors, body mass index (BMI), height, weight, and player exposure were likely insignificant. Furthermore, there is evidence supporting age as a possible risk factor for hamstring injury in soccer players [5,25,26]. The literature has shown conflicting evidence on the influence of muscle strength, although one high-quality prospective study found that hamstring and quadriceps strength deficits are weak risk factors for a hamstring injury and some authors question their clinical relevance. More recent studies have found a significant relationship between the isokinetic strength of knee flexors (hamstring) and extensors at 60°/s, as well as in the flexor/extensor angular velocity ratio of 60°/s, with the occurrence of injury in soccer [27]. Another prospective study of 146 professional players found that soccer players with low levels of isokinetic hamstring strength and low hamstring–quadriceps strength ratio have an increased risk of acute hamstring injury [28]. In general, a decreased hamstring strength variable increases the risk of acute injury [29].

Quadriceps peak torque was considered a risk factor in a recent systematic review and meta-analysis. This marker was found in four soccer-related studies. Consequently, quadriceps peak torque may be considered a predictive factor in soccer, although more soccer-specific studies are needed for a conclusive statement [30]. A large number of potential predictors of hamstring injuries have been investigated, but there is currently insufficient evidence to draw conclusions. The most important factors are age [11,30,31,32], previous injury [11,30,31,33,34], increased quadriceps torque [11,30,32], asymmetry of eccentric hamstring strength [11,30], lower body stiffness [11,35,36], and single-leg bridge test [11,30]. Furthermore, according to the book Return to Play in Football: An Evidence-based Approach [11], the psychological component or position in the game is also a relevant factor.

Regarding ML techniques, there are a few studies found in the literature that discuss explainable machine learning. From these studies, it was determined that the most popular techniques are classifiers [37,38,39], post-explanatory ML techniques [40,41,42,43,44], and feature selection [42,45]. From the studies using feature selection, it was observed that it can improve the performance of prediction models and make the results more interpretable. In this regard, one study proposed a method to identify the most important features for the assessment of joint-space-narrowing progression in patients with knee osteoarthritis [46]. Another study employed fuzzy logic to combine multiple feature importance scores, which were used for the identification and interpretation of knee osteoarthrosis risk factors. The presented methodology was able to select a subset of risk factors that increased the accuracy of several ML models compared to popular selection techniques [45], indicating that feature selection is a good option when the goal is to enhance the explainability of the results.

## 3. Soccer Player Injury Classification Architecture

**Figure 1:**
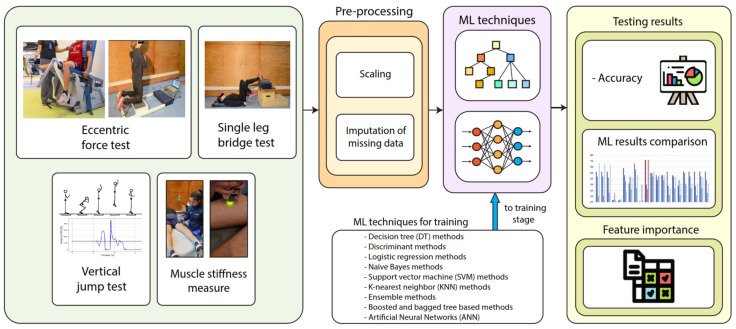
Proposed architecture for the soccer player injury classification based on muscle biomechanical analysis.

In this study, we present an injury classification architecture based on four distinct biomechanical measures derived from professional soccer players. The architecture is depicted in Figure 1 and comprises various stages, including the collection of the dataset for biomechanical testing, pre-processing, classification, and the final classification results. We provide a detailed explanation of each stage of the proposed architecture in the following sections.

### 3.1. Dataset for Biomechanical Tests

In this work, 110 male professional soccer players were evaluated to build the proposed dataset, corresponding to three of the main first-division soccer teams in Chile. For each player, we considered the player’s age, weight, height, and several biomechanical test results, anthropometric measurements, and positions of the players within the team, which, in total, represent 19 different features. All the experiments and evaluations were conducted by kinesiologists at the Biomechanics Laboratory of the Innovation Center, located within the MEDS Clinic in Santiago, Chile. The exclusion criteria encompassed injuries within the last three months and a body mass index (BMI) below 24. All participants provided informed consent before participation, and adherence to the exclusion and inclusion criteria was verified before data acquisition. The data matrix was provided anonymously by the MEDS clinic, thus guaranteeing the confidentiality of the data.

#### Biomechanical Tests

**Figure 2:**
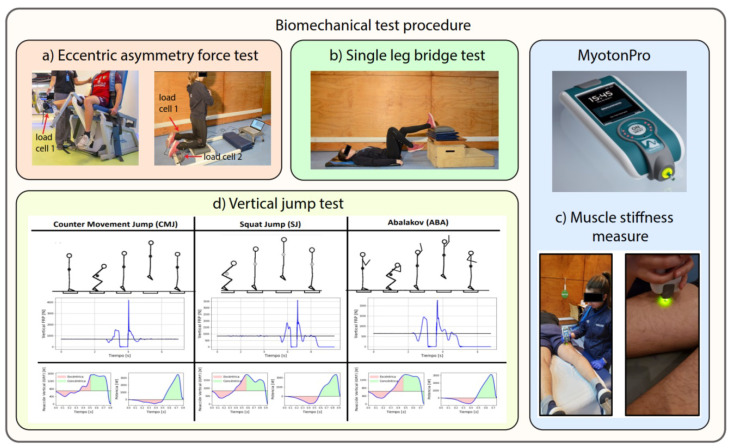
Biomechanical test procedure.

- Eccentric asymmetry force test (Nordic Hamstring): The participants assume a kneeling position with aligned hips and trunk support (see Figure 2a). An assistant or, in this case, load cells, is responsible for securing the heels, ensuring continual contact with the ground during the exercise. Load cells are utilized to measure the eccentric activation of the hamstring muscles. This test yields two parameters: the maximum right hamstring eccentric force (N) and the maximum left hamstring eccentric force (N).
- Eccentric force test (Maximum Force Quadriceps): The participants use a quadriceps chair (Figure 2a) connected to load cells to measure the eccentric activation of the quadriceps muscles. This test delivers the parameters of the right quadriceps muscle’s maximal eccentric force (N) and the left quadriceps muscle’s maximal force (N).
- Single-leg bridge test: This clinical test assesses susceptibility to hamstring injury. The participant is instructed to lie on the floor supine, with the heel of the designated leg placed inside a 60 cm high box. With hands crossed over the chest, the subject must push with the heel to elevate the glutes off the ground. Each repetition requires the participant to touch the ground before raising the glutes again without resting (see Figure 2b). This test yields the number of repetitions for the right leg and the number of repetitions for the left leg.
- Muscle stiffness measure (Myotonometry): This technique involves an objective and non-invasive digital palpation method for superficial skeletal muscles. The measurement explicitly targets the hamstring muscles (see Figure 2c) and is conducted using a MyotonPRO device. The parameter obtained for both extremities includes the S–stiffness (N/m), which reflects the resistance to force or contraction that induces structural or tissue deformation.
- Vertical jump test (Bosco test): This series of vertical jumps serves to evaluate various aspects, including morphophysiological characteristics (muscle fiber type), functional attributes (height and mechanical jump power), and neuromuscular features (utilization of elastic energy and myotatic reflex, fatigue resistance) of the lower limb extensor muscles based on the attained jump height and mechanical power in different types of vertical jumps. The Bosco test employs three jumps on a force platform. The execution of these jumps can be observed in Figure 2d, encompassing data from the countermovement jump (both two-legged and one-legged), squat jump (both two-legged and one-legged), and Abalakov (both bipodal and unipodal) jump.

### 3.2. Pre-Processing

In this study, a pre-processing stage was implemented to ensure the integrity and reliability of the data for the subsequent machine learning classification stage. Scaling and imputation techniques were applied to effectively handle missing data and standardize the variables. Scaling was employed to standardize the force measures in relation to the body weight of each player. This adjustment aimed to ensure a fair evaluation by mitigating the dominance of participant-specific body variations, thus promoting an unbiased analysis. Furthermore, to address the issue of missing data, zeros were used to fill in the gaps, given that not all players were available for certain tests due to various reasons such as prior injuries. This approach was pivotal in preserving the data’s completeness and preventing potential biases during the analysis. Overall, these pre-processing methods significantly contributed to preparing the data for comprehensive analysis and interpretation.

### 3.3. Classification

Once the data were pre-processed, we trained several ML algorithms to evaluate and compare their classification performance. For this, a feature matrix with dimensions of 110×19 was assembled, with 110 rows representing the participants and 19 columns signifying the various biomechanical test results, anthropometric measurements, and positions within the team (forward, defender, goalkeeper, or midfielder). Each sample of the dataset was categorized into two classes. Class 0 represents no lower limb muscle injuries during the playing season and class 1 represents lower limb muscle injuries during the playing season. The database was constructed by physical therapists, kinesiologists, and traumatologists from the MEDS clinic. It was this multidisciplinary team that reviewed the injury history and placed the labels according to the hamstring injuries sustained in the last year (last playing season).

To find the best possible classifier for our dataset, a total of 35 machine learning (ML) techniques were implemented, including decision tree (DT) methods, discriminant methods, logistic regression, naive Bayes, support vector machine (SVM), K-nearest neighbor (KNN), ensemble methods, boosted and bagged trees, artificial neural networks (ANNs), and XGBoost. We present a brief description and the configuration of each of the proposed ML models in Table 1 and Table 2, respectively. Then, we used a cross-validation evaluation technique with k = 10 folds, where the dataset was divided into ten parts, allowing each of the proposed models to be trained and evaluated ten times with different combinations of training and test data. This means that for each of the ten experiments, we used 90% of the data for training and 10% of the data for testing. This ensures a robust evaluation, avoiding bias due to a single partition. Additionally, this method optimizes model generalization by using multiple splits, handles the inherent variability of the data, and helps identify overfitting or underfitting.

In addition, the feature importance analysis obtained using the XGBoost model was utilized to identify the most important and differentiating characteristics of the dataset. For this, multiple iterations were conducted, considering the best-performing characteristics from N = 30 iterations. The evaluation metrics, including the cross-validation and confusion matrix, were derived to validate the classification performance. These influential characteristics, deemed as injury biomarkers, were used as the focus of the analysis in this study.

### 3.4. Most Important Features

To obtain the features that contribute most to class differentiation, several iterations were performed, and the features were considered in N iterations (where N was set to 30). For this, the feature importance module of XGBoost was used.

### 3.5. Results

**Figure 3:**
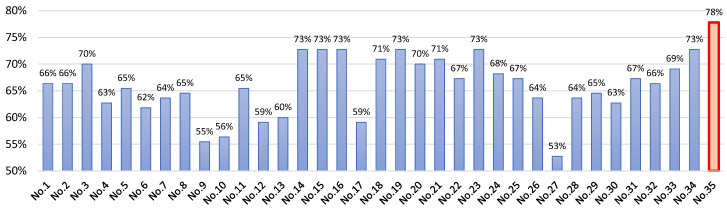
Comparison of ML models’ testing accuracies (with ten k-folds).

The testing results of applying 35 ML algorithms are shown in Table 1, along with the configuration and description of each model. The accuracy values of each model are visualized in Figure 3, where it can be seen that the best performance was obtained using the XGBoost technique, achieving an accuracy of 78%, followed by the SVM, decision tree, KNN, and logistic regression kernel techniques, which achieved accuracies of higher than 70%.

**Figure 4:**
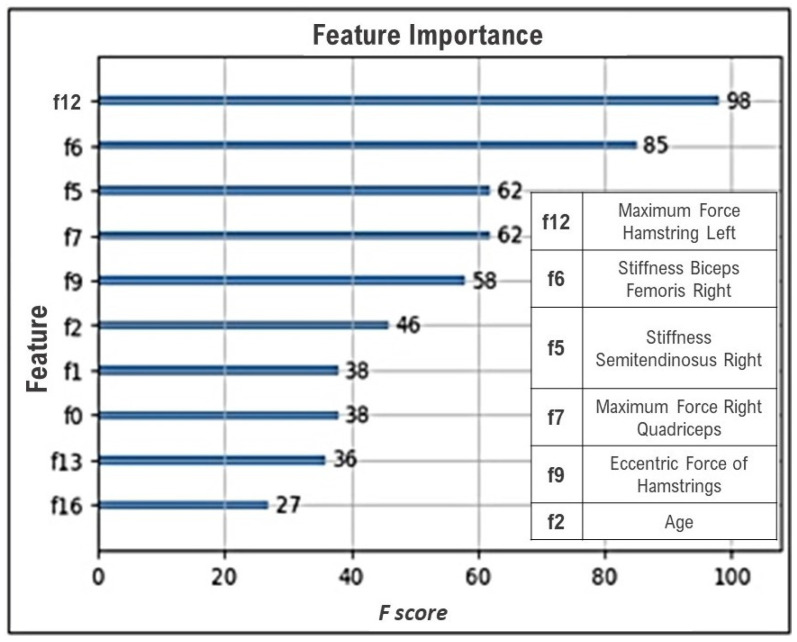
Feature importance.

Table 3 shows the most important characteristics obtained by XGBoost, where the variable with the greatest weight in the classification, or the one that was repeated the most during 30 iterations, was the maximum left hamstring strength, followed by the right biceps femoris stiffness and semitendinosus stiffness. Figure 4 shows an example of the feature importance graph of the best-performing model, displaying the same variables as in Table 3, indicating that the most important feature is the maximum hamstring force.

## 4. Discussion

Contemporary research primarily focuses on optimizing the functionality of intricate machine learning models, often neglecting their capacity for explanation. Consequently, healthcare professionals encounter challenges in comprehending these models and struggle to place trust in their outputs [44,47,48,49]. Thus, there is a growing demand for advanced ML detection and prediction models that can aid doctors in early and precise disease diagnosis [2,44,48]. Hence, both model performance and explainability are important in facilitating sound decision making.

Studies have demonstrated that in research aiming to provide interpretability to results, the most commonly employed ML techniques include random forests, decision trees, K-nearest neighbors (KNN), and support vector machines. These simpler models are typically favored when the emphasis lies on generating more comprehensible and interpretable models [49]. Notably, the construction of these models is informed by scientific evidence, suggestions based on various data sources, and expert opinions [14]. Some of these models were implemented in this work, and their performance results are shown in Figure 3. The best performance was obtained with model Nº 35, corresponding to the XGboost model, which achieved an accuracy of 78%. This result aligns with another study that applied several ML techniques and also found that XGBoost exhibited the best performance [50].

Figure 4 highlights the most influential iteration, showcasing the maximum force of the left hamstring as the variable with the highest weight. The most significant features contributing to the best results are detailed in Table 3. The findings suggest that the maximum muscle strength of the hamstrings and the stiffness of the same muscle are the key variables that distinguish the groups and could serve as effective predictors for injury prevention. However, there is conflicting evidence concerning the influence of muscle strength. A high-quality prospective study indicated that hamstring and quadriceps strength deficits were weak risk factors for hamstring strain injuries (HSIs), casting doubt on their clinical significance [25]. Conversely, a recent systematic review focused on strength training as the primary approach to prevention [5]. Nonetheless, to establish a definitive statement, further specific studies within this sport are required [5]. Regarding team differences, aside from the significance of quadriceps strength, as mentioned earlier, disparities in player age have also been identified. Evidence shows that age is a potential risk factor for hamstring injuries in soccer players [5,25]. Hence, it becomes essential to assess whether differences in the number of injuries per team are attributable to this factor and review the type of training programs each group employs, considering the potential impact of these differences on performance.

It is worth mentioning that in developing this study, we made great efforts to build and process an important and unique database that allows soccer player injury classification based on muscle biomechanical analysis. In an attempt to broaden the scope of our work and make our evaluation as fair as possible, we tested and evaluated 35 different machine learning and deep learning algorithms, which cover the majority of the most efficient classification algorithms such as SVM, K-NN, decision trees, bagged trees, logistic regression, neural networks, and XGBoost. Additionally, we used cross-validation with k = 10 folds in an attempt to make the evaluation as fair and unbiased as possible. In future work, we will focus on the search and evaluation of potential new machine learning algorithms that are specialized for this particular application.

## 5. Conclusions

A notable disparity exists between academic research outcomes and their practical implementation in medical practice. Medical professionals hesitate to rely on decisions generated by opaque black box models lacking comprehensive and easily understandable explanations [51]. Consequently, ML techniques utilized in clinical settings typically avoid complex models in favor of simpler and more interpretable ones, albeit at the expense of precision or intricacy. In this context, applying the XGBoost technique instills confidence in the outcomes and offers a more interpretable perspective from a medical standpoint. The results from this technique indicate that favorable precision can be achieved by incorporating scientific evidence, suggestions based on diverse data sources, and expert opinions, thereby enhancing the trustworthiness of the results for doctors and trainers. Moreover, the obtained results strongly align with the existing literature, although additional specific studies within this sport remain imperative to establish a definitive statement. As already known, the prediction of hamstring injuries in soccer using machine learning techniques is a constantly evolving area of research. In this sense, it would be ideal to consider additional variables such as anthropometric measurements, training levels, nutritional conditions, physiological variables, biometric data, medical images, on-field performance data, and even genetic variables. In terms of ML analytics, the integration of machine learning models with fuzzy logic can be investigated to create hybrid systems. These systems can leverage the capabilities of machine learning and the interpretability of fuzzy logic to improve accuracy and model understanding. The development of systems that use fuzzy logic to translate the rules extracted by machine learning models into a language understandable by sports professionals would facilitate both the interpretation of model decisions and preventative action.

## Figures and Tables

**Table 1:** ML models, configurations, and description—Part 1.

No.	Model Name	Model Configuration	Model Description
No. 1	Tree	100 splits	
No. 2	Tree	20 splits	A flowchart-like structure where an internal node represents a feature, a branch represents a decision rule, and each leaf node represents an outcome.
No. 3	Tree	4 splits	
No. 4	Linear discriminant	Full covariance structure	A statistical technique for binary and multiclass classification, which finds the linear combination of features that best separates classes.
No. 5	Quadratic discriminant	Full covariance structure	A method similar to linear discriminant analysis, but it assumes that the features follow a Gaussian distribution and estimates the covariance between the classes.
No. 6	Binary GLM Logistic Regression	Binomial distribution	Logistic regression with binary outcomes for estimating the probability of a binary outcome using a logistic function.
No. 7	Efficient Logistic Regression	L2 regularization, alpha = 0.001, one-vs-one coding	A regression analysis similar to binary logistic regression but implemented efficiently to handle large datasets or high-dimensional data.
No. 8	Efficient Linear SVM	L2 regularization, alpha = 0.001, one-vs-one coding	A supervised machine learning algorithm used for classification and regression analysis, which finds a hyperplane that best separates classes.
No. 9	Gaussian Naive Bayes	Gaussian distribution	A probabilistic classifier, which assumes that the presence of a particular feature in a class is unrelated to the presence of other features.
No. 10	Kernel Naive Bayes	Normal kernel, data standardization	A version of the naive Bayes classifier that can handle non-linear classification using kernel methods, transforming data into higher dimensions.
No. 11	Linear SVM	Linear kernel, one-vs-one coding, data standardization	A supervised machine learning algorithm used for classification, which finds a hyperplane that best separates classes in a linearly separable dataset.
No. 12	Quadratic SVM	Quadratic kernel, one-vs-one coding, data standardization	An extension of the SVM algorithm that uses a quadratic kernel to handle non-linearly separable data by mapping it into a higher-dimensional space.
No. 13	Cubic SVM	Cubic kernel, one-vs-one coding, data standardization	An extension of the SVM algorithm that uses a cubic kernel to handle highly non-linearly separable data by mapping it into an even higher-dimensional space.
No. 14	Fine Gaussian SVM	Kernel scale = 1.6, one-vs-one coding, data standardization	An SVM with a fine Gaussian kernel, suitable for datasets requiring high precision and accuracy.
No. 15	Medium Gaussian SVM	kernel scale = 6.5, one-vs-one coding, data standardization	An SVM with a medium Gaussian kernel, suitable for datasets with moderate complexity and dimensionality.
No. 16	Coarse Gaussian SVM	Kernel scale = 26, one-vs-one coding, data standardization	An SVM with a coarse Gaussian kernel, suitable for datasets with lower complexity and dimensionality.

**Table 2:** ML models, configurations, and descriptions—Part 2.

No.	Model Name	Model Configuration	Model Description
No. 17	Fine KNN	Number of neighbors = 1, Euclidean distance	A non-parametric classification algorithm, that classifies a data point based on the majority vote of its neighbors with a fine-tuned distance metric.
No. 18	Medium KNN	Number of neighbors = 10, Euclidean distance	A non-parametric classification algorithm that classifies a data point based on the majority vote of its neighbors with a moderately adjusted distance metric.
No. 19	Coarse KNN	Number of neighbors = 100, Euclidean distance	A non-parametric classification algorithm that classifies a data point based on the majority vote of its neighbors with a roughly adjusted distance metric.
No. 20	Cosine KNN	Number of neighbors = 10, Euclidean distance	A variation of the K-nearest neighbor algorithm that computes the cosine similarity between data points to measure their similarity.
No. 21	Cubic KNN	Number of neighbors = 10, Euclidean distance	A non-parametric classification algorithm that classifies a data point based on the majority vote of its neighbors with a cubic distance metric.
No. 22	Weighted KNN	Number of neighbors = 10, Euclidean distance	A variant of the K-nearest neighbor algorithm that assigns weights to the contributions of the neighbors based on their distances.
No. 23	Boosted Trees with AdaBoost ensemble	Decision tree learner, maximum splits = 20, learning rate=0.1	An ensemble learning method that constructs a strong classifier by combining multiple weak classifiers such as decision trees using the AdaBoost algorithm.
No. 24	Bagged trees with bag ensemble	Decision tree learner, maximum splits = 109, number of learners = 30	An ensemble learning technique that combines multiple models such as decision trees to improve classification accuracy and stability.
No. 25	Subspace discriminant ensemble	Discriminant learner, number of learners = 30, subspace dimension = 10	An ensemble approach that combines multiple discriminant analysis models to improve the classification performance of the system.
No. 26	Subspace KNN ensemble	Subspace ensemble method, decision tree learner, number of learners = 30, learning rate = 0.1	An ensemble learning technique that combines multiple K-nearest neighbor models operating in different subspaces to improve classification accuracy.
No. 27	RUSBoosted Trees	RUSBoost ensemble method, decision tree learner, number of learners = 30, learning rate = 0.1	It is a variant of the AdaBoost algorithm that incorporates random under-sampling to address class imbalance, particularly in binary classification problems.
No. 28	Neural Network	1 layer, 10 neurons, 1k iterations	A network of interconnected nodes inspired by the structure of the human brain, capable of learning complex patterns and relationships within data.
No. 29	Neural Network	1 layer, 25 neurons, 1k iterations	
No. 30	Neural Network	1 layer, 100 neurons, 1k iterations	
No. 31	Neural Network	2 layers, 10 neuron, 1k iterations	
No. 32	Neural Network	3 layers, 10 neurons, 1k iterations	
No. 33	SVM Kernel	SVM learner, lambda regularization = 0.01, one-vs-one coding, iteration limit = 1000	A variant of the SVM algorithm that uses kernel methods to handle non-linear data by transforming it into a higher-dimensional space.
No. 34	Logistic regression kernel	Logistic regression learner, lambda regularization = 0.01, one-vs-one coding,	A variant of logistic regression that uses kernel methods to handle non-linear data.
No. 35	XGBoost	learning rate = 0.3, L2 regularization alpha = 0.001, sampling method = uniform	An optimized gradient-boosting library designed for speed and performance, effective for classification and regression.

**Table 3:** Most important features (most repeated over 30 iterations) obtained by XGBoost.

Feature	Number of Repetitions
Maximum Force Hamstring Left	28
Stiffness Biceps Femoris Right	28
Stiffness Semitendinosus Right	24
Maximum Force Right Quadriceps	21
Eccentric Force of Hamstrings	17
Age	16

## Data Availability

The data are not publicly available due to MEDS Clinic restrictions.

## References

[1] Baroni B.M., Ruas C.V., Ribeiro-Alvares J.B., Pinto R.S. (2020). Hamstring-to-quadriceps torque ratios of professional male soccer players: A systematic review. J. Strength Cond. Res..

[2] Lee G., Nho K., Kang B., Sohn K.A., Kim D. (2019). Predicting Alzheimer’s disease progression using multi-modal deep learning approach. Sci. Rep..

[3] Cumps E., Verhagen E., Annemans L., Meeusen R. (2008). Injury rate and socioeconomic costs resulting from sports injuries in Flanders: Data derived from sports insurance statistics 2003. Br. J. Sport. Med..

[4] Calderón-Díaz M., Ulloa-Jiménez R., Saavedra C., Salas R. (2021). Wavelet-based semblance analysis to determine muscle synergy for different handstand postures of Chilean circus athletes. Comput. Methods Biomech. Biomed. Eng..

[5] Rosado-Portillo A., Chamorro-Moriana G., Gonzalez-Medina G., Perez-Cabezas V. (2021). Acute hamstring injury prevention programs in eleven-a-side football players based on physical exercises: Systematic review. J. Clin. Med..

[6] Grazioli R., Lopez P., Andersen L.L., Machado C.L.F., Pinto M.D., Cadore E.L., Pinto R.S. (2019). Hamstring rate of torque development is more affected than maximal voluntary contraction after a professional soccer match. Eur. J. Sport Sci..

[7] Crema M.D., Guermazi A., Tol J.L., Niu J., Hamilton B., Roemer F.W. (2016). Acute hamstring injury in football players: Association between anatomical location and extent of injury—a large single-center MRI report. J. Sci. Med. Sport.

[8] Ekstrand J., Hägglund M., Kristenson K., Magnusson H., Waldén M. (2013). Fewer ligament injuries but no preventive effect on muscle injuries and severe injuries: An 11-year follow-up of the UEFA Champions League injury study. Br. J. Sport. Med..

[9] Szymski D., Krutsch V., Achenbach L., Gerling S., Pfeifer C., Alt V., Krutsch W., Loose O. (2022). Epidemiological analysis of injury occurrence and current prevention strategies on international amateur football level during the UEFA Regions Cup 2019. Arch. Orthop. Trauma Surg..

[10] Biz C., Nicoletti P., Baldin G., Bragazzi N.L., Crimì A., Ruggieri P. (2021). Hamstring strain injury (HSI) prevention in professional and semi-professional football teams: A systematic review and meta-analysis. Int. J. Environ. Res. Public Health.

[11] Musahl V., Karlsson J., Krutsch W., Mandelbaum B.R., Espregueira-Mendes J., d’Hooghe P. (2018). Return to Play in Football: An Evidence-Based Approach.

[12] Cos F., Cos M.Á., Buenaventura L., Pruna R., Ekstrand J. (2010). Modelos de análisis para la prevención de lesiones en el deporte. Estudio epidemiológico de lesiones: El modelo Union of European Football Associations en el fútbol. Apunts. Med. De L’Esport.

[13] Halilaj E., Rajagopal A., Fiterau M., Hicks J.L., Hastie T.J., Delp S.L. (2018). Machine learning in human movement biomechanics: Best practices, common pitfalls, and new opportunities. J. Biomech..

[14] Handelman G., Kok H., Chandra R., Razavi A., Lee M., Asadi H. (2018). eDoctor: Machine learning and the future of medicine. J. Intern. Med..

[15] Tseng P.Y., Chen Y.T., Wang C.H., Chiu K.M., Peng Y.S., Hsu S.P., Chen K.L., Yang C.Y., Lee O.K.S. (2020). Prediction of the development of acute kidney injury following cardiac surgery by machine learning. Crit. Care.

[16] Pan S.L., Zhang S. (2020). From fighting COVID-19 pandemic to tackling sustainable development goals: An opportunity for responsible information systems research. Int. J. Inf. Manag..

[17] Ramkumar P.N., Karnuta J.M., Haeberle H.S., Owusu-Akyaw K.A., Warner T.S., Rodeo S.A., Nwachukwu B.U., Williams III R.J. (2021). Association between preoperative mental health and clinically meaningful outcomes after osteochondral allograft for cartilage defects of the knee: A machine learning analysis. Am. J. Sport. Med..

[18] Jamaludin A., Lootus M., Kadir T., Zisserman A., Urban J., Battié M.C., Fairbank J., McCall I. (2017). Automation of reading of radiological features from magnetic resonance images (MRIs) of the lumbar spine without human intervention is comparable with an expert radiologist. Eur. Spine J..

[19] Mak W.K., Bin Abd Razak H.R., Tan H.C.A. (2019). Which Patients Require a Contralateral Total Knee Arthroplasty Within 5 Years of Index Surgery?. J. Knee Surg..

[20] Masís S. (2021). Interpretable Machine Learning with Python: Learn to Build Interpretable High-Performance Models with Hands-On Real-World Examples.

[21] Chen H., Michalopoulos G., Subendran S., Yang R., Quinn R., Oliver M., Butt Z., Wong A. Interpretability of ml models for health data-a case study. Proceedings of the First Annual International Workshop on Interpretability: Methodologies and Algorithms—IMA 2019.

[22] Linardatos P., Papastefanopoulos V., Kotsiantis S. (2020). Explainable ai: A review of machine learning interpretability methods. Entropy.

[23] Brownlee J. (2016). XGBoost With Python: Gradient Boosted Trees with XGBoost and Scikit-Learn.

[24] Van Beijsterveldt A., van de Port I.G., Vereijken A., Backx F. (2013). Risk factors for hamstring injuries in male soccer players: A systematic review of prospective studies. Scand. J. Med. Sci. Sport..

[25] Hägglund M., Waldén M., Ekstrand J. (2006). Previous injury as a risk factor for injury in elite football: A prospective study over two consecutive seasons. Br. J. Sport. Med..

[26] Arnason A., Sigurdsson S.B., Gudmundsson A., Holme I., Engebretsen L., Bahr R. (2004). Risk factors for injuries in football. Am. J. Sport. Med..

[27] Namazi P., Zarei M., Hovanloo F., Abbasi H. (2019). The association between the isokinetic muscle strength and lower extremity injuries in young male football players. Phys. Ther. Sport.

[28] Lee J.W., Mok K.M., Chan H.C., Yung P.S., Chan K.M. (2018). Eccentric hamstring strength deficit and poor hamstring-to-quadriceps ratio are risk factors for hamstring strain injury in football: A prospective study of 146 professional players. J. Sci. Med. Sport.

[29] Green B., Bourne M.N., van Dyk N., Pizzari T. (2020). Recalibrating the risk of hamstring strain injury (HSI): A 2020 systematic review and meta-analysis of risk factors for index and recurrent hamstring strain injury in sport. Br. J. Sport. Med..

[30] Freckleton G., Pizzari T. (2013). Risk factors for hamstring muscle strain injury in sport: A systematic review and meta-analysis. Br. J. Sport. Med..

[31] Fousekis K., Tsepis E., Poulmedis P., Athanasopoulos S., Vagenas G. (2011). Intrinsic risk factors of non-contact quadriceps and hamstring strains in soccer: A prospective study of 100 professional players. Br. J. Sport. Med..

[32] Henderson G., Barnes C.A., Portas M.D. (2010). Factors associated with increased propensity for hamstring injury in English Premier League soccer players. J. Sci. Med. Sport.

[33] Fyfe J.J., Opar D.A., Williams M.D., Shield A.J. (2013). The role of neuromuscular inhibition in hamstring strain injury recurrence. J. Electromyogr. Kinesiol..

[34] Warren P., Gabbe B.J., Schneider-Kolsky M., Bennell K.L. (2010). Clinical predictors of time to return to competition and of recurrence following hamstring strain in elite Australian footballers. Br. J. Sport. Med..

[35] Blackburn J.T., Norcross M.F. (2014). The effects of isometric and isotonic training on hamstring stiffness and anterior cruciate ligament loading mechanisms. J. Electromyogr. Kinesiol..

[36] Watsford M.L., Murphy A.J., McLachlan K.A., Bryant A.L., Cameron M.L., Crossley K.M., Makdissi M. (2010). A prospective study of the relationship between lower body stiffness and hamstring injury in professional Australian rules footballers. Am. J. Sport. Med..

[37] Amaral J.L., Sancho A.G., Faria A.C., Lopes A.J., Melo P.L. (2020). Differential diagnosis of asthma and restrictive respiratory diseases by combining forced oscillation measurements, machine learning and neuro-fuzzy classifiers. Med. Biol. Eng. Comput..

[38] Song X., Gu F., Wang X., Ma S., Wang L. (2021). Interpretable Recognition for Dementia Using Brain Images. Front. Neurosci..

[39] Sabol P., Sinčák P., Hartono P., Kočan P., Benetinová Z., Blichárová A., Verbóová L., Štammová E., Sabolová-Fabianová A., Jašková A. (2020). Explainable classifier for improving the accountability in decision-making for colorectal cancer diagnosis from histopathological images. J. Biomed. Inform..

[40] García-Pérez P., Lozano-Milo E., Landin M., Gallego P.P. (2020). Machine Learning unmasked nutritional imbalances on the medicinal plant Bryophyllum sp. cultured in vitro. Front. Plant Sci..

[41] Apostolopoulos I.D., Groumpos P.P., Apostolopoulos D.J. (2021). Advanced fuzzy cognitive maps: State-space and rule-based methodology for coronary artery disease detection. Biomed. Phys. Eng. Express.

[42] Juang C.F., Wen C.Y., Chang K.M., Chen Y.H., Wu M.F., Huang W.C. (2021). Explainable fuzzy neural network with easy-to-obtain physiological features for screening obstructive sleep apnea-hypopnea syndrome. Sleep Med..

[43] Ding L., Zhang X.y., Wu D.y., Liu M.l. (2021). Application of an extreme learning machine network with particle swarm optimization in syndrome classification of primary liver cancer. J. Integr. Med..

[44] El-Sappagh S., Alonso J.M., Islam S.R., Sultan A.M., Kwak K.S. (2021). A multilayer multimodal detection and prediction model based on explainable artificial intelligence for Alzheimer’s disease. Sci. Rep..

[45] Kokkotis C., Ntakolia C., Moustakidis S., Giakas G., Tsaopoulos D. (2022). Explainable machine learning for knee osteoarthritis diagnosis based on a novel fuzzy feature selection methodology. Phys. Eng. Sci. Med..

[46] Ntakolia C., Kokkotis C., Moustakidis S., Tsaopoulos D. (2021). Identification of most important features based on a fuzzy ensemble technique: Evaluation on joint space narrowing progression in knee osteoarthritis patients. Int. J. Med. Inform..

[47] Burkart N., Huber M.F. (2021). A survey on the explainability of supervised machine learning. J. Artif. Intell. Res..

[48] Bucholc M., Ding X., Wang H., Glass D.H., Wang H., Prasad G., Maguire L.P., Bjourson A.J., McClean P.L., Todd S. (2019). A practical computerized decision support system for predicting the severity of Alzheimer’s disease of an individual. Expert Syst. Appl..

[49] Das D., Ito J., Kadowaki T., Tsuda K. (2019). An interpretable machine learning model for diagnosis of Alzheimer’s disease. PeerJ.

[50] Khan I.U., Aslam N., AlShedayed R., AlFrayan D., AlEssa R., AlShuail N.A., Al Safwan A. (2022). A proactive attack detection for heating, ventilation, and air conditioning (HVAC) system using explainable extreme gradient boosting model (XGBoost). Sensors.

[51] Calderón-Díaz M., Serey-Castillo L.J., Vallejos-Cuevas E.A., Espinoza A., Salas R., Macías-Jiménez M.A. (2023). Detection of variables for the diagnosis of overweight and obesity in young Chileans using machine learning techniques. Procedia Comput. Sci..

